# Synthesis and crystal structure of [Pd{C_6_H_4_(CH_2_NHCH_2_Ph)-2-κ^2^ *C*,*N*}(μ-I)]_2_

**DOI:** 10.1107/S2056989017014281

**Published:** 2017-10-06

**Authors:** Delia Bautista, Sergio J. Benitez-Benitez

**Affiliations:** a SAI, Universidad de Murcia, Murcia 30100, Spain; b Departamento Química Inorgánica, Universidad de Murcia, Murcia 30071, Spain

**Keywords:** crystal structure, iodide bridge, *C*,*N*-cyclo­palladated complex

## Abstract

The binuclear title complex, di-μ-iodido-bis(­{2-[(benzylamino-κ*N*)meth­yl]phenyl-κ*C* ^1^}palladium(II)), [Pd_2_I_2_(C_14_H_14_N)_2_], was prepared by reaction of [Pd{C_6_H_4_(CH_2_NHCH_2_Ph)-2}(μ-OAc)]_2_ with NaI. It crystallizes with one discrete mol­ecule in the asymmetric unit. The mol­ecule presents an iodide-bridged dimeric structure with a *cisoid* arrangement with respect to the *C*,*N*-cyclo­palladated ligands. Both Pd^II^ atoms have a slightly distorted square-planar coordination environment. Weak inter­molecular contacts of the type C—H⋯Pd seem to have a significant influence on the arrangement of the mol­ecules along the *b* axis in the crystal.

## Abstract 2

The binuclear mol­ecules of the title complex show weak inter­molecular C—H⋯Pd inter­actions, whereas the amine N—H functional groups are not involved in hydrogen bonding.

## Chemical context   

Cyclo­palladated complexes (Dupont *et al.*, 2005 ▸) have important applications in homogeneous catalysis (Bravo *et al.*, 2002 ▸), as chiral resolving agents (Gugger *et al.*, 2008 ▸), drugs (Cutillas *et al.*, 2013 ▸), or new materials (Jayabharathi *et al.*, 2011 ▸).
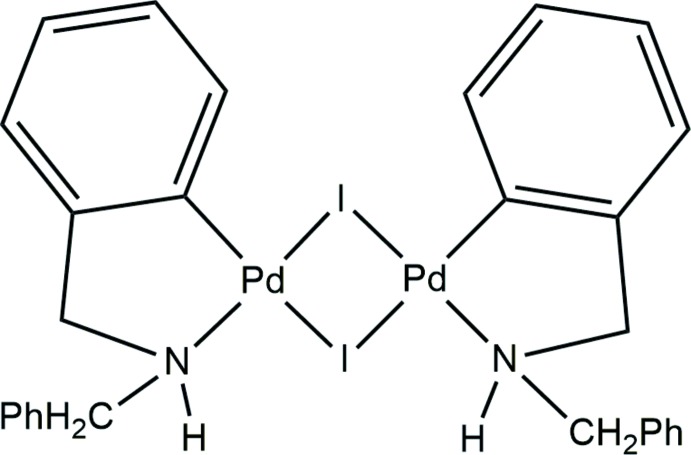


Over the past few years, our group has been inter­ested in the synthesis, reactivity and applications in organic synthesis of orthopalladated derivatives of di­benzyl­amine. We have reported the first general method for the cyclo­palladation of primary and secondary amines by using Pd(OAc)_2_. The acetato-bridged complexes were transformed into the corres­ponding halido-bridged complexes by anion metathesis reactions, which were used for further reactivity studies (Vicente *et al.*, 1997 ▸).

Herein we report the synthesis and crystal structure of a iodido-bridged complex [Pd{C_6_H_4_(CH_2_NHCH_2_Ph)-2}(μ-I)]_2_. This is a rare example of a cyclo­palladated complex containing bridging iodido ligands and one of the few C^N-cyclo­palladated iodido-bridged complexes characterized by X-ray diffraction.

## Structural commentary   

**Figure 1:**
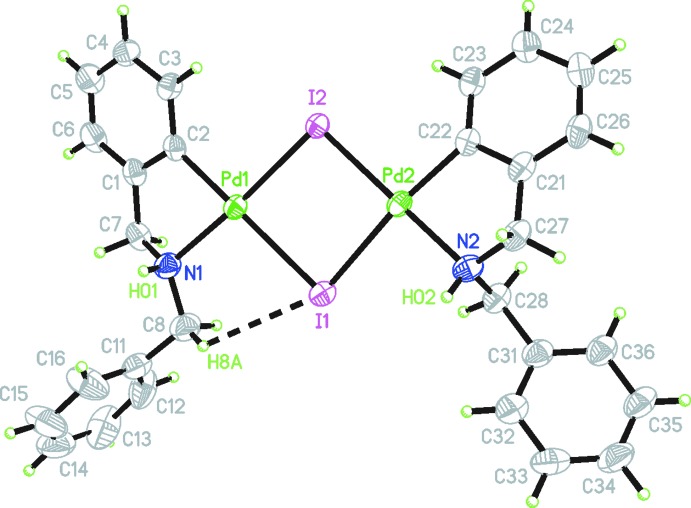
The mol­ecular structure of the title complex, with displacement ellipsoids at the 50% probability level. The black dashed line indicates the intra­molecular C—H⋯I hydrogen bond (see Table 2 ▸ for numerical details).

The complex crystallizes in the centrosymmetric monoclinic space group *P*2_1_/*n* with one mol­ecule in the asymmetric unit. The mol­ecular structure can be described as a nearly planar dipalladium subunit of the type (C–N)Pd(μ-I)_2_Pd(C–N) (Fig. 1 ▸). Both palladium atoms adopt a slightly distorted square-planar coordination environment, the mean deviations of the Pd—N—C—I—I planes being larger for Pd2 (0.0868 Å) than for Pd1 (0.0301 Å). The highest deviation from the average coordination plane occurs for C22 (0.1261 Å). The more distorted square-planar geometry of Pd2 is further evidenced by the smaller dihedral angle between the planes N1—Pd1—C2 and I1—Pd1—I2 [5.53 (16)°] compared to that of N2—Pd2—C22 and I1—Pd2—I2 [8.29 (16)°]. The structural differences around both Pd^II^ atoms are consistent with the presence of two N—H stretching bands at 3261 and 3201 cm^−1^ in the infrared spectrum of the solid.

In contrast to the unsymmetrical dimers with a *cisoid* arrangement of the ligands observed in the title compound [Pd{C_6_H_4_(CH_2_NHCH_2_Ph)-_2_}(μ-I)]_2_, the di­bromido analogue [Pd{C_6_H_4_(CH_2_NHCH_2_Ph)-2}(μ-Br)]_2_ (Vicente *et al.*, 1999 ▸) shows a centrosymmetric dimer with a *transoid* disposition of the chelating ligands involving the amino groups.

Owing to the *cisoid* arrangement of the C,N-cyclo­palladated ligands, one of the iodine atoms of the Pd_2_I_2_ unit is *trans* to two carbon atoms (I1) whereas the other is *trans* to two nitro­gen atoms (I2). Consequently, the Pd—I bond lengths of the I atoms *trans* to N [2.5959 (5) and 2.5801 (4) Å] are shorter than those of the I atoms *trans* to C [2.7504 (5) and 2.7030 (5) Å] because of the greater *trans* influence of the aryl ligands compared to that of the amino ligands. Similar values for these bond lengths and also for the C—Pd [1.986 (5), 1.991 (4) Å] and N—Pd [2.104 (4), 2.809 (4) Å] bond lengths have been found in the five structures of iodido-bridged cyclo­palladated complexes reported so far (see *Database survey*). Selected torsion angles are collated in Table 1 ▸.

One of the methyl­enic hydrogen atoms of the cyclo­palladated di­benzyl­amine moiety coordinating to Pd1 participates in the formation of a non-classical intra­molecular C—H⋯I hydrogen bond (Fig. 1 ▸, Table 2 ▸).

## Supra­molecular features   

**Figure 2:**
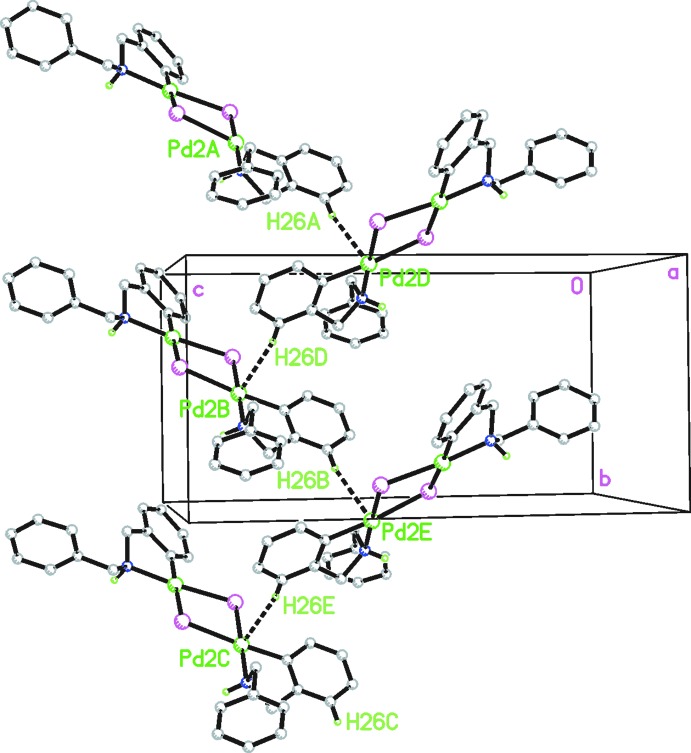
A view of the mol­ecular packing of the title compound. Dotted lines indicate C—H⋯Pd contacts. H atoms not involved in the inter­actions have been omitted for clarity.

There are no hydrogen-bonding inter­actions involving the two NH groups. The most remarkable inter­molecular inter­action observed in the crystal structure is a weak hydrogen bond between the arylic hydrogen placed in position 3 of the phenyl­ene ring attached to Pd2 (H16) and the Pd2 atom of the adjacent mol­ecule. This inter­action gives rise to the formation of a chain arrangement of mol­ecules along the *b* axis (Fig. 2 ▸). Although the Pd—H bond length [2.760 (2) Å] is slightly shorter than the sum of the van der Waals radii of Pd and H (2.83 Å) (Bondi, 1964 ▸), it seems to direct the arrangement of the mol­ecules in the crystal structure. In this context it is inter­esting to compare the arrangement of the mol­ecules in this complex with that of the di­bromido analogue [Pd{C_6_H_4_(CH_2_NHCH_2_Ph)-_2_}(μ-Br)]_2_ (Vicente *et al.*, 1999 ▸), which is formed by stacking of nearly co-planar complex palladium dimers, where the empty space is filled by solvent mol­ecules (CH_2_Cl_2_). Such a disposition appears to be normal in dimeric halido-bridging cyclo­metalated complexes of *d* ^8^ elements (Aullón *et al.*, 1998 ▸) and hence contrasts with the unusual structure observed in the title compound [Pd{C_6_H_4_(CH_2_NHCH_2_Ph)-2}(μ-I)]_2_.

## Database survey   

A search in the Cambridge Structural Database (Groom *et al.*, 2016 ▸) gave only six reports of binuclear iodido-bridged orthopalladated complexes with different bidentate C—N ligands: *N,N*-di­methyl­benzyl­amine (Gül & Nelson, 2000 ▸), azo­benzene derivatives (Ghedini *et al.*, 1999 ▸; Crispini *et al.*, 1993 ▸), imines (Praefcke *et al.*, 1995 ▸) and ferrocenyloxazoline derivatives (Donde & Overman, 1999 ▸; Anderson *et al.*, 2005 ▸), with the following bond lengths ranges: Pd—I: 2.591 (3)–2.581 (5) Å (*trans* to N), 2.724 (4)– 2.694 (5) Å (*trans* to C); C—Pd: 1.964 (8)–2.113 (2) Å; N—Pd, 2.008 (8)–2.065 (5) Å].

## Synthesis and crystallization   

To a suspension of the complex [Pd{C_6_H_4_(CH_2_NHCH_2_Ph)-2}(μ-OAc)]_2_ (Vicente *et al.*, 1999 ▸) (800 mg, 1.106 mmol) in acetone (30 ml) solid NaI (1000 mg, 6.022 mmol) was added and the resulting mixture was stirred for 3 h. The solution was filtered through a plug of MgSO_4_, and the filtrate was concentrated to *ca* 5 ml. Diethyl ether was added (25 ml), the solvent was partially removed (to *ca* 5 ml), and *n*-pentane was added (25 ml) to precipitate the title complex as an orange solid, which was collected and air-dried. Single crystals of the compound suitable for X-ray analysis were obtained by slow diffusion of *n*-pentane into a solution of the compound in CHCl_3_ at room temperature. Yield 845 mg, 0.983 mmol, 89%. Analysis calculated for C_28_H_28_I_2_N_2_Pd_2_ (859.2): C, 39.11; H, 3.26; N, 3.26. Found: C, 38.80; H, 3.21; N, 3.21. IR (Nujol, cm^−1^): *ν*(N—H) = 3261, 3201. ^1^H NMR (CDCl_3_, 400 MHz): *d* = 3.83–3.95 (*m*, 2H, CH_2_), 4.18 (*s*, *b*, 1H, NH), 4.23–4.29 (*m*, 1H, CH_2_), 4.65 (*d*, *b*, 1H, CH_2_, ^2^ *J*_HH_ = 12.9 Hz), 6.83–6.87 (*m*, 1H, CH, C_6_H_4_), 6.92–7.00 (*m*, 2H, CH, C_6_H_4_), 7.32–7.41 (*m*, 5H, Ph), 7.67 (*s*, *b*, 1H, C_6_H_4_, ^3^ *J*_HH_ = 7.6 Hz). ^13^C{^1^H} NMR (CDCl_3_, 75 MHz): *d* = 57.4 (*s*, CH_2_), 59.4 (*s*, CH_2_), 122.6 (*s*, CH, C_6_H_4_), 124.6 (*s*, CH, C_6_H_4_), 126.4 (*s*, CH, C_6_H_4_), 128.6 (*s*, *p*-CH, Ph), 129.1 (*s*, *m*-CH, Ph), 129.3 (*s*, *o*-CH, Ph), 135.6 (*s*, *i*-C, Ph), 138.5 (*s*, CH, C_6_H_4_), 147.8 (*s*, C, C_6_H_4_), 150.6 (*s*, C, C_6_H_4_).

## Refinement   

Crystal data, data collection and structure refinement details are summarized in Table 3 ▸. C and N atoms were subjected to DELU commands (Sheldrick, 2015 ▸), and five reflections were omitted from the final refinement due to poor agreement between measured and calculated intensities. All H atoms associated with C atoms could be located in difference-Fourier maps. However, they were relocated at geometrically idealized positions and were allowed to ride on the parent atoms with C—H = 0.95 Å (aromatic) and 0.99 Å (CH_2_) and *U*_iso_(H) = 1.2*U*_eq_(C). Hydrogen atoms bound to N atoms were discernible from a difference-Fourier map and were subsequently refined with N—H distance restraints [target value 0.87 (2) Å].

## Supplementary Material

Crystal structure: contains datablock(s) I. DOI: 10.1107/S2056989017014281/wm5411sup1.cif

Structure factors: contains datablock(s) I. DOI: 10.1107/S2056989017014281/wm5411Isup3.hkl

CCDC reference: 1577834

Additional supporting information:  crystallographic information; 3D view; checkCIF report

## Figures and Tables

**Table 1:** Selected torsion angles (°)

C2—C1—C7—N1	23.5 (6)	C22—C21—C27—N2	29.2 (6)
C7—N1—C8—C11	−54.9 (7)	C27—N2—C28—C31	67.4 (5)

**Table 2:** Hydrogen-bond geometry (Å, °)

*D*—H⋯*A*	*D*—H	H⋯*A*	*D*⋯*A*	*D*—H⋯*A*
C8—H8*A*⋯I1	0.99	2.94	3.444 (6)	113

**Table 3:** Experimental details

Crystal data	
Chemical formula	[Pd_2_I_2_(C_14_H_14_N)_2_]
*M*_r_	859.12
Crystal system, space group	Monoclinic, *P*2_1_/*n*
Temperature (K)	100
*a*, *b*, *c* (Å)	14.2201 (12), 9.9787 (7), 19.4205 (13)
β (°)	90.200 (2)
*V* (Å^3^)	2755.7 (4)
*Z*	4
Radiation type	Mo *K*α
μ (mm^−1^)	3.57
Crystal size (mm)	0.15 × 0.10 × 0.04

Data collection	
Diffractometer	Bruker D8 QUEST
Absorption correction	Multi-scan (*SADABS*; Krause *et al.*, 2015 ▸)
*T*_min_, *T*_max_	0.788, 0.928
No. of measured, independent and observed [*I* > 2σ(*I*)] reflections	70523, 5768, 5148
*R*_int_	0.034
(sin θ/λ)_max_ (Å^−1^)	0.630

Refinement	
*R*[*F* ^2^ > 2σ(*F* ^2^)], *wR*(*F* ^2^), *S*	0.031, 0.084, 1.03
No. of reflections	5768
No. of parameters	315
No. of restraints	319
H-atom treatment	H atoms treated by a mixture of independent and constrained refinement
Δρ_max_, Δρ_min_ (e Å^−3^)	2.45, −0.65

Computer programs: *APEX2* and *SAINT* (Bruker, 2013 ▸), *SHELXS97* and *SHELXTL* (Sheldrick, 2008 ▸) and *SHELXL2013* (Sheldrick, 2015 ▸).

## supplementary crystallographic information

### Crystal data

**Table:**

[Pd_2_I_2_(C_14_H_14_N)_2_]	*F*(000) = 1632
*M**_r_* = 859.12	*D*_x_ = 2.071 Mg m^−^^3^
Monoclinic, *P*2_1_/*n*	Mo *K*α radiation, λ = 0.71073 Å
*a* = 14.2201 (12) Å	Cell parameters from 9807 reflections
*b* = 9.9787 (7) Å	θ = 2.3–26.6°
*c* = 19.4205 (13) Å	µ = 3.57 mm^−^^1^
β = 90.200 (2)°	*T* = 100 K
*V* = 2755.7 (4) Å^3^	Lath, orange
*Z* = 4	0.15 × 0.10 × 0.04 mm

### Data collection

**Table:**

Bruker D8 QUEST diffractometer	5768 independent reflections
Radiation source: high brilliance microfocus sealed tube	5148 reflections with *I* > 2σ(*I*)
Detector resolution: 10.4167 pixels mm^-1^	*R*_int_ = 0.034
ω–scans	θ_max_ = 26.6°, θ_min_ = 2.1°
Absorption correction: multi-scan (*SADABS*; Krause *et al.*, 2015)	*h* = −17→17
*T*_min_ = 0.788, *T*_max_ = 0.928	*k* = −12→12
70523 measured reflections	*l* = −24→24

### Refinement

**Table:**

Refinement on *F*^2^	319 restraints
Least-squares matrix: full	Hydrogen site location: mixed
*R*[*F*^2^ > 2σ(*F*^2^)] = 0.031	H atoms treated by a mixture of independent and constrained refinement
*wR*(*F*^2^) = 0.084	*w* = 1/[σ^2^(*F*_o_^2^) + (0.0439*P*)^2^ + 10.4728*P*] where *P* = (*F*_o_^2^ + 2*F*_c_^2^)/3
*S* = 1.03	(Δ/σ)_max_ = 0.001
5768 reflections	Δρ_max_ = 2.45 e Å^−^^3^
315 parameters	Δρ_min_ = −0.65 e Å^−^^3^

### Special details

**Table:**

Geometry. All esds (except the esd in the dihedral angle between two l.s. planes) are estimated using the full covariance matrix. The cell esds are taken into account individually in the estimation of esds in distances, angles and torsion angles; correlations between esds in cell parameters are only used when they are defined by crystal symmetry. An approximate (isotropic) treatment of cell esds is used for estimating esds involving l.s. planes. Least-squares planes (x,y,z in crystal coordinates) and deviations from them (* indicates atom used to define plane) 6.1807 (0.0096) x - 8.2633 (0.0047) y + 6.8462 (0.0164) z = 3.8899 (0.0181) * 0.0632 (0.0014) Pd1 * 0.0071 (0.0020) N1 * -0.0439 (0.0021) C2 * -0.0312 (0.0014) I1 * 0.0048 (0.0016) I2 Rms deviation of fitted atoms = 0.0373 7.4752 (0.0099) x - 7.3667 (0.0058) y + 8.1731 (0.0140) z = 6.1885 (0.0155) Angle to previous plane (with approximate esd) = 8.325 ( 0.117 ) * 0.0427 (0.0013) Pd2 * 0.1010 (0.0020) N2 * -0.1261 (0.0020) C22 * -0.0908 (0.0015) I1 * 0.0732 (0.0015) I2 Rms deviation of fitted atoms = 0.0911
Refinement. The hydrogens atoms at NH were refined with DFIX, others as rigid.

### Fractional atomic coordinates and isotropic or equivalent isotropic displacement parameters (Å^2^)

**Table:**

	*x*	*y*	*z*	*U*_iso_*/*U*_eq_	
Pd1	0.47515 (2)	0.70685 (3)	1.00161 (2)	0.02890 (9)	
Pd2	0.34849 (2)	0.47810 (3)	0.87459 (2)	0.02862 (9)	
I1	0.30166 (2)	0.58520 (3)	0.99762 (2)	0.03447 (9)	
I2	0.49727 (2)	0.62609 (3)	0.87564 (2)	0.03242 (9)	
N1	0.4641 (3)	0.7879 (4)	1.1013 (2)	0.0380 (9)	
H01	0.494 (4)	0.729 (5)	1.127 (2)	0.045 (16)*	
N2	0.2317 (3)	0.3510 (4)	0.8740 (2)	0.0370 (9)	
H02	0.231 (4)	0.317 (6)	0.9151 (15)	0.048 (17)*	
C1	0.5929 (3)	0.9138 (4)	1.0531 (2)	0.0332 (9)	
C2	0.5942 (3)	0.8107 (4)	1.0039 (2)	0.0286 (9)	
C3	0.6740 (3)	0.7940 (4)	0.9634 (2)	0.0327 (9)	
H3	0.677255	0.722141	0.931399	0.039*	
C4	0.7488 (4)	0.8826 (5)	0.9699 (3)	0.0406 (11)	
H4	0.803004	0.870965	0.942083	0.049*	
C5	0.7454 (4)	0.9875 (6)	1.0164 (3)	0.0513 (14)	
H5	0.796102	1.049342	1.019104	0.062*	
C6	0.6688 (4)	1.0024 (5)	1.0585 (3)	0.0437 (12)	
H6	0.667352	1.072824	1.091399	0.052*	
C7	0.5084 (4)	0.9232 (5)	1.0990 (2)	0.0364 (10)	
H7A	0.527604	0.951407	1.145846	0.044*	
H7B	0.463367	0.989848	1.080537	0.044*	
C8	0.3699 (4)	0.7860 (6)	1.1331 (3)	0.0505 (13)	
H8A	0.347701	0.691992	1.134865	0.061*	
H8B	0.326034	0.836288	1.103039	0.061*	
C11	0.3647 (3)	0.8443 (5)	1.2049 (3)	0.0426 (11)	
C12	0.3065 (4)	0.9528 (6)	1.2173 (4)	0.0653 (18)	
H12	0.275421	0.996211	1.180096	0.078*	
C13	0.2939 (5)	0.9979 (8)	1.2842 (4)	0.083 (2)	
H13	0.252076	1.070199	1.292786	0.100*	
C14	0.3408 (5)	0.9395 (8)	1.3378 (4)	0.0750 (19)	
H14	0.332063	0.971284	1.383422	0.090*	
C15	0.4000 (6)	0.8356 (8)	1.3252 (3)	0.081 (2)	
H15	0.434133	0.795865	1.362036	0.097*	
C16	0.4105 (5)	0.7881 (7)	1.2594 (3)	0.0683 (19)	
H16	0.450879	0.713792	1.251593	0.082*	
C21	0.3024 (3)	0.3022 (4)	0.7638 (2)	0.0338 (9)	
C22	0.3629 (3)	0.4095 (4)	0.7789 (2)	0.0294 (9)	
C23	0.4149 (3)	0.4648 (5)	0.7258 (2)	0.0345 (10)	
H23	0.455206	0.538695	0.734839	0.041*	
C24	0.4089 (4)	0.4137 (5)	0.6598 (3)	0.0412 (11)	
H24	0.445559	0.451778	0.623915	0.049*	
C25	0.3493 (4)	0.3067 (5)	0.6458 (3)	0.0431 (11)	
H25	0.345393	0.271395	0.600469	0.052*	
C26	0.2962 (3)	0.2519 (5)	0.6974 (3)	0.0384 (10)	
H26	0.254951	0.179341	0.687572	0.046*	
C27	0.2478 (4)	0.2432 (5)	0.8225 (3)	0.0397 (11)	
H27A	0.186886	0.207751	0.805648	0.048*	
H27B	0.283548	0.168638	0.843681	0.048*	
C28	0.1430 (3)	0.4257 (5)	0.8621 (3)	0.0406 (11)	
H28A	0.141848	0.459322	0.814148	0.049*	
H28B	0.141125	0.504176	0.893271	0.049*	
C31	0.0568 (4)	0.3397 (5)	0.8743 (3)	0.0424 (11)	
C32	0.0347 (4)	0.3003 (7)	0.9398 (3)	0.0618 (17)	
H32	0.072487	0.328740	0.977520	0.074*	
C33	−0.0430 (5)	0.2189 (8)	0.9513 (4)	0.076 (2)	
H33	−0.057735	0.190253	0.996600	0.091*	
C34	−0.0981 (4)	0.1800 (7)	0.8969 (3)	0.0585 (15)	
H34	−0.151242	0.124423	0.904654	0.070*	
C35	−0.0775 (4)	0.2200 (6)	0.8331 (3)	0.0485 (13)	
H35	−0.116842	0.193593	0.795804	0.058*	
C36	0.0005 (3)	0.2995 (5)	0.8205 (3)	0.0442 (12)	
H36	0.014989	0.326193	0.774793	0.053*	

### Atomic displacement parameters (Å^2^)

**Table:**

	*U*^11^	*U*^22^	*U*^33^	*U*^12^	*U*^13^	*U*^23^
Pd1	0.02632 (17)	0.02705 (17)	0.03331 (18)	−0.00158 (13)	−0.00086 (13)	−0.00183 (13)
Pd2	0.02629 (17)	0.02353 (17)	0.03602 (18)	−0.00306 (12)	−0.00117 (13)	0.00140 (13)
I1	0.02959 (16)	0.03506 (17)	0.03877 (17)	−0.00318 (12)	0.00233 (12)	0.00046 (12)
I2	0.03033 (16)	0.03417 (16)	0.03276 (16)	−0.00964 (11)	0.00015 (11)	−0.00282 (12)
N1	0.034 (2)	0.039 (2)	0.041 (2)	−0.0039 (17)	0.0036 (17)	−0.0084 (18)
N2	0.035 (2)	0.032 (2)	0.044 (2)	−0.0045 (17)	0.0042 (18)	0.0047 (18)
C1	0.044 (3)	0.027 (2)	0.029 (2)	−0.0038 (19)	−0.0059 (18)	0.0070 (17)
C2	0.036 (2)	0.024 (2)	0.026 (2)	−0.0005 (17)	−0.0055 (16)	0.0069 (16)
C3	0.034 (2)	0.025 (2)	0.039 (2)	−0.0056 (17)	−0.0025 (18)	0.0040 (18)
C4	0.040 (3)	0.043 (3)	0.039 (3)	−0.016 (2)	0.002 (2)	0.006 (2)
C5	0.063 (3)	0.050 (3)	0.041 (3)	−0.034 (3)	0.001 (2)	0.003 (2)
C6	0.065 (3)	0.034 (3)	0.033 (2)	−0.020 (2)	−0.003 (2)	0.0038 (19)
C7	0.050 (3)	0.027 (2)	0.032 (2)	0.000 (2)	−0.003 (2)	−0.0013 (18)
C8	0.038 (3)	0.057 (3)	0.056 (3)	−0.001 (2)	0.008 (2)	−0.010 (3)
C11	0.035 (3)	0.041 (3)	0.052 (3)	−0.004 (2)	0.008 (2)	−0.008 (2)
C12	0.045 (3)	0.064 (4)	0.087 (4)	0.014 (3)	−0.019 (3)	−0.025 (3)
C13	0.052 (4)	0.085 (5)	0.113 (5)	0.021 (3)	−0.006 (4)	−0.057 (4)
C14	0.062 (4)	0.093 (5)	0.070 (4)	−0.015 (4)	0.030 (3)	−0.025 (4)
C15	0.094 (6)	0.101 (6)	0.047 (3)	0.016 (4)	0.022 (4)	0.011 (4)
C16	0.087 (5)	0.067 (4)	0.051 (3)	0.033 (4)	0.019 (3)	0.012 (3)
C21	0.030 (2)	0.025 (2)	0.046 (2)	0.0001 (17)	−0.0045 (19)	0.0013 (19)
C22	0.029 (2)	0.023 (2)	0.036 (2)	0.0022 (16)	−0.0049 (17)	0.0008 (17)
C23	0.036 (2)	0.027 (2)	0.041 (2)	−0.0030 (18)	−0.0046 (19)	0.0029 (19)
C24	0.050 (3)	0.036 (3)	0.038 (2)	−0.003 (2)	−0.005 (2)	0.005 (2)
C25	0.050 (3)	0.039 (3)	0.040 (3)	0.000 (2)	−0.009 (2)	−0.004 (2)
C26	0.037 (2)	0.028 (2)	0.051 (3)	−0.0006 (19)	−0.010 (2)	−0.006 (2)
C27	0.036 (2)	0.030 (2)	0.053 (3)	−0.0081 (19)	−0.002 (2)	−0.001 (2)
C28	0.033 (2)	0.038 (3)	0.051 (3)	−0.0006 (19)	−0.002 (2)	−0.001 (2)
C31	0.035 (2)	0.041 (3)	0.052 (3)	0.001 (2)	0.008 (2)	−0.005 (2)
C32	0.043 (3)	0.092 (5)	0.051 (3)	−0.014 (3)	0.002 (3)	0.000 (3)
C33	0.052 (4)	0.118 (6)	0.058 (3)	−0.022 (4)	0.019 (3)	0.008 (4)
C34	0.032 (3)	0.071 (4)	0.073 (4)	−0.010 (3)	0.011 (3)	−0.004 (3)
C35	0.030 (2)	0.046 (3)	0.069 (3)	−0.002 (2)	0.005 (2)	−0.006 (3)
C36	0.032 (2)	0.043 (3)	0.057 (3)	0.001 (2)	0.001 (2)	−0.004 (2)

### Geometric parameters (Å, º)

**Table:**

Pd1—C2	1.986 (5)	C13—C14	1.364 (9)
Pd1—N1	2.104 (4)	C13—H13	0.9500
Pd1—I2	2.5959 (5)	C14—C15	1.358 (9)
Pd1—I1	2.7504 (5)	C14—H14	0.9500
Pd2—C22	1.991 (4)	C15—C16	1.373 (8)
Pd2—N2	2.090 (4)	C15—H15	0.9500
Pd2—I2	2.5801 (4)	C16—H16	0.9500
Pd2—I1	2.7030 (5)	C21—C26	1.387 (6)
N1—C8	1.477 (7)	C21—C22	1.404 (6)
N1—C7	1.491 (6)	C21—C27	1.502 (7)
N1—H01	0.884 (19)	C22—C23	1.387 (6)
N2—C28	1.482 (6)	C23—C24	1.382 (6)
N2—C27	1.487 (7)	C23—H23	0.9500
N2—H02	0.87 (2)	C24—C25	1.390 (7)
C1—C6	1.399 (7)	C24—H24	0.9500
C1—C2	1.404 (6)	C25—C26	1.370 (7)
C1—C7	1.501 (7)	C25—H25	0.9500
C2—C3	1.392 (7)	C26—H26	0.9500
C3—C4	1.388 (6)	C27—H27A	0.9900
C3—H3	0.9500	C27—H27B	0.9900
C4—C5	1.383 (8)	C28—C31	1.516 (7)
C4—H4	0.9500	C28—H28A	0.9900
C5—C6	1.373 (8)	C28—H28B	0.9900
C5—H5	0.9500	C31—C32	1.369 (7)
C6—H6	0.9500	C31—C36	1.375 (7)
C7—H7A	0.9900	C32—C33	1.390 (8)
C7—H7B	0.9900	C32—H32	0.9500
C8—C11	1.512 (8)	C33—C34	1.368 (8)
C8—H8A	0.9900	C33—H33	0.9500
C8—H8B	0.9900	C34—C35	1.336 (8)
C11—C16	1.361 (7)	C34—H34	0.9500
C11—C12	1.384 (7)	C35—C36	1.386 (7)
C12—C13	1.388 (9)	C35—H35	0.9500
C12—H12	0.9500	C36—H36	0.9500
			
C2—Pd1—N1	81.12 (17)	C13—C12—H12	120.1
C2—Pd1—I2	94.40 (13)	C14—C13—C12	120.7 (6)
N1—Pd1—I2	174.83 (12)	C14—C13—H13	119.6
C2—Pd1—I1	174.71 (12)	C12—C13—H13	119.6
N1—Pd1—I1	97.21 (11)	C15—C14—C13	119.4 (6)
I2—Pd1—I1	87.023 (13)	C15—C14—H14	120.3
C22—Pd2—N2	82.55 (17)	C13—C14—H14	120.3
C22—Pd2—I2	96.72 (12)	C14—C15—C16	120.1 (7)
N2—Pd2—I2	177.54 (12)	C14—C15—H15	120.0
C22—Pd2—I1	170.83 (12)	C16—C15—H15	120.0
N2—Pd2—I1	92.69 (12)	C11—C16—C15	121.9 (6)
I2—Pd2—I1	88.352 (14)	C11—C16—H16	119.1
Pd2—I1—Pd1	88.605 (13)	C15—C16—H16	119.1
Pd2—I2—Pd1	94.767 (14)	C26—C21—C22	120.5 (4)
C8—N1—C7	114.1 (4)	C26—C21—C27	122.2 (4)
C8—N1—Pd1	116.8 (3)	C22—C21—C27	117.3 (4)
C7—N1—Pd1	106.7 (3)	C23—C22—C21	118.4 (4)
C8—N1—H01	101 (4)	C23—C22—Pd2	128.0 (3)
C7—N1—H01	114 (4)	C21—C22—Pd2	113.1 (3)
Pd1—N1—H01	104 (4)	C24—C23—C22	120.9 (4)
C28—N2—C27	113.1 (4)	C24—C23—H23	119.6
C28—N2—Pd2	111.8 (3)	C22—C23—H23	119.6
C27—N2—Pd2	108.6 (3)	C23—C24—C25	120.0 (5)
C28—N2—H02	109 (4)	C23—C24—H24	120.0
C27—N2—H02	110 (4)	C25—C24—H24	120.0
Pd2—N2—H02	104 (4)	C26—C25—C24	120.2 (5)
C6—C1—C2	120.1 (5)	C26—C25—H25	119.9
C6—C1—C7	122.3 (4)	C24—C25—H25	119.9
C2—C1—C7	117.6 (4)	C25—C26—C21	120.1 (4)
C3—C2—C1	119.0 (4)	C25—C26—H26	120.0
C3—C2—Pd1	128.4 (3)	C21—C26—H26	120.0
C1—C2—Pd1	112.6 (3)	N2—C27—C21	107.9 (4)
C4—C3—C2	119.8 (5)	N2—C27—H27A	110.1
C4—C3—H3	120.1	C21—C27—H27A	110.1
C2—C3—H3	120.1	N2—C27—H27B	110.1
C5—C4—C3	120.9 (5)	C21—C27—H27B	110.1
C5—C4—H4	119.6	H27A—C27—H27B	108.4
C3—C4—H4	119.6	N2—C28—C31	112.3 (4)
C6—C5—C4	120.0 (5)	N2—C28—H28A	109.1
C6—C5—H5	120.0	C31—C28—H28A	109.1
C4—C5—H5	120.0	N2—C28—H28B	109.1
C5—C6—C1	120.0 (5)	C31—C28—H28B	109.1
C5—C6—H6	120.0	H28A—C28—H28B	107.9
C1—C6—H6	120.0	C32—C31—C36	119.2 (5)
N1—C7—C1	107.5 (4)	C32—C31—C28	119.8 (5)
N1—C7—H7A	110.2	C36—C31—C28	121.0 (5)
C1—C7—H7A	110.2	C31—C32—C33	120.1 (6)
N1—C7—H7B	110.2	C31—C32—H32	119.9
C1—C7—H7B	110.2	C33—C32—H32	119.9
H7A—C7—H7B	108.5	C34—C33—C32	119.7 (6)
N1—C8—C11	115.4 (5)	C34—C33—H33	120.1
N1—C8—H8A	108.4	C32—C33—H33	120.1
C11—C8—H8A	108.4	C35—C34—C33	120.3 (6)
N1—C8—H8B	108.4	C35—C34—H34	119.8
C11—C8—H8B	108.4	C33—C34—H34	119.8
H8A—C8—H8B	107.5	C34—C35—C36	120.8 (6)
C16—C11—C12	118.2 (5)	C34—C35—H35	119.6
C16—C11—C8	122.2 (5)	C36—C35—H35	119.6
C12—C11—C8	119.5 (5)	C31—C36—C35	119.9 (5)
C11—C12—C13	119.7 (6)	C31—C36—H36	120.1
C11—C12—H12	120.1	C35—C36—H36	120.1
			
C6—C1—C2—C3	3.4 (6)	C26—C21—C22—C23	0.9 (7)
C7—C1—C2—C3	−176.0 (4)	C27—C21—C22—C23	179.3 (4)
C6—C1—C2—Pd1	−175.9 (4)	C26—C21—C22—Pd2	173.1 (4)
C7—C1—C2—Pd1	4.7 (5)	C27—C21—C22—Pd2	−8.5 (5)
C1—C2—C3—C4	−3.0 (7)	C21—C22—C23—C24	−1.3 (7)
Pd1—C2—C3—C4	176.1 (4)	Pd2—C22—C23—C24	−172.2 (4)
C2—C3—C4—C5	0.2 (8)	C22—C23—C24—C25	0.8 (8)
C3—C4—C5—C6	2.3 (9)	C23—C24—C25—C26	0.2 (8)
C4—C5—C6—C1	−1.9 (8)	C24—C25—C26—C21	−0.7 (8)
C2—C1—C6—C5	−0.9 (7)	C22—C21—C26—C25	0.1 (7)
C7—C1—C6—C5	178.5 (5)	C27—C21—C26—C25	−178.2 (5)
C8—N1—C7—C1	−168.6 (4)	C28—N2—C27—C21	90.4 (5)
Pd1—N1—C7—C1	−38.0 (4)	Pd2—N2—C27—C21	−34.3 (4)
C6—C1—C7—N1	−155.9 (4)	C26—C21—C27—N2	−152.4 (4)
C2—C1—C7—N1	23.5 (6)	C22—C21—C27—N2	29.2 (6)
C7—N1—C8—C11	−54.9 (7)	C27—N2—C28—C31	67.4 (5)
Pd1—N1—C8—C11	179.6 (4)	Pd2—N2—C28—C31	−169.6 (3)
N1—C8—C11—C16	−64.5 (8)	N2—C28—C31—C32	69.7 (6)
N1—C8—C11—C12	120.1 (6)	N2—C28—C31—C36	−109.8 (6)
C16—C11—C12—C13	−2.3 (9)	C36—C31—C32—C33	1.0 (8)
C8—C11—C12—C13	173.3 (6)	C28—C31—C32—C33	−178.5 (6)
C11—C12—C13—C14	2.4 (10)	C31—C32—C33—C34	−1.1 (10)
C12—C13—C14—C15	−0.4 (12)	C32—C33—C34—C35	0.1 (11)
C13—C14—C15—C16	−1.6 (12)	C33—C34—C35—C36	1.0 (10)
C12—C11—C16—C15	0.3 (11)	C32—C31—C36—C35	0.0 (8)
C8—C11—C16—C15	−175.1 (7)	C28—C31—C36—C35	179.5 (5)
C14—C15—C16—C11	1.7 (13)	C34—C35—C36—C31	−1.0 (9)

### Hydrogen-bond geometry (Å, º)

**Table:**

*D*—H···*A*	*D*—H	H···*A*	*D*···*A*	*D*—H···*A*
C8—H8*A*···I1	0.99	2.94	3.444 (6)	113

## References

[bb1] Anderson, C. E., Donde, Y., Douglas, C. J. & Overman, L. E. (2005). *J. Org. Chem.* **70**, 648–657. 10.1021/jo048490r 15651813

[bb2] Aullón, G., Ujaque, G., Lledós, A., Álvarez, S. & Alemany, P. (1998). *Inorg. Chem.* **37**, 804–813.

[bb3] Bondi, A. (1964). *J. Phys. Chem.* **68**, 441–451.

[bb4] Bravo, J., Cativiela, C., Navarro, R. & Urriolabeitia, E. P. (2002). *J. Organomet. Chem.* **650**, 157–172.

[bb5] Bruker (2013). *APEX2* and *SAINT*. Bruker AXS Inc. Madison Wisconsin, USA.

[bb6] Crispini, A., Ghedini, M. & Neve, F. (1993). *J. Organomet. Chem.* **448**, 241–245.

[bb7] Cutillas, N., Yellol, G. S., de Haro, C., Vicente, C., Rodríguez, V. & Ruiz, J. (2013). *Coord. Chem. Rev.* **257**, 2784–2797.

[bb8] Donde, Y. & Overman, L. E. (1999). *J. Am. Chem. Soc.* **121**, 2933–2934.

[bb9] Dupont, J., Consorti, C. S. & Spencer, J. (2005). *Chem. Rev.* **105**, 2527–2571. 10.1021/cr030681r 15941221

[bb10] Ghedini, M., Pucci, D., Crispini, A., Aiello, I., Barigelletti, F., Gessi, A. & Francescangeli, O. (1999). *Appl. Organomet. Chem.* **13**, 565–581.

[bb11] Groom, C. R., Bruno, I. J., Lightfoot, M. P. & Ward, S. C. (2016). *Acta Cryst.* B**72**, 171–179. 10.1107/S2052520616003954 PMC4822653 27048719

[bb12] Gugger, P. A., Hockless, D. C., Kilah, N. L., Mayadunne, R. C. & Wild, S. B. (2008). *Tetrahedron Asymmetry*, **19**, 1810–1812.

[bb13] Gül, N. & Nelson, J. H. (2000). *Organometallics*, **19**, 91–104.

[bb14] Jayabharathi, J., Thanikachalam, V., Saravanan, K. & Srinivasan, N. (2011). *J. Fluoresc.* **21**, 507–519. 10.1007/s10895-010-0737-7 20953824

[bb15] Krause, L., Herbst-Irmer, R., Sheldrick, G. M. & Stalke, D. (2015). *J. Appl. Cryst.* **48**, 3–10. 10.1107/S1600576714022985 PMC4453166 26089746

[bb16] Praefcke, K., Diele, S., Pickardt, J., Gündogman, B., Nütz, U. & Singer, D. (1995). *Liq. Cryst.* **18**, 857–865.

[bb17] Sheldrick, G. M. (2008). *Acta Cryst.* A**64**, 112–122. 10.1107/S0108767307043930 18156677

[bb18] Sheldrick, G. M. (2015). *Acta Cryst.* C**71**, 3–8.

[bb19] Vicente, J., Saura-Llamas, I., Palin, M. G., Jones, P. G. & Ramírez de Arellano, M. C. (1997). *Organometallics*, **16**, 826–833.

[bb20] Vicente, J., Saura-Llamas, I., Turpín, J., Ramírez de Arellano, M. C. & Jones, P. G. (1999). *Organometallics*, **18**, 2683–2693.

